# Influencing Factors and Improvement Path of Academic Engagement among College Students in the Context of Epidemic Prevention and Control

**DOI:** 10.3390/ijerph191912939

**Published:** 2022-10-10

**Authors:** Xiangju Yin, Yiming Huang, Xin Zhang, Yuqian Chen, Mingyue Wang, Hongwei Qian

**Affiliations:** 1 School of Emergency Management, Henan Polytechnic University, Jiaozuo 454000, China; 2 Emergency Science and Engineering Research Center, Henan Polytechnic University, Jiaozuo 454003, China

**Keywords:** health behaviors, perceived social support, learning engagement, online teaching

## Abstract

Objective: The implementation of online teaching in the context of epidemic prevention and control has had an impact on the learning engagement of college students to some extent. This study aims to investigate the mechanisms that influence perceived social support and health behaviors on learning engagement, so as to make college students more focused on their studies by improving their physical and mental health as well as their ability to perceive social support. Methods: A total of 538 college students from Henan Province, China, were studied using the Perceived Social Support Scale, Health Behavior Scale and Learning Engagement Scale, and the data were analyzed by IBM SPSS Amos 26.0 software (IBM SPSS Inc., Chicago, IL, USA). Results: (1) The level of health behavior among college students was positively correlated with perceived social support ability (β = 0.289, *p* < 0.001); both perceived social support and health behaviors predicted college students’ learning engagement significantly (β = 0.200, *p* < 0.01; β = 0.406, *p* < 0.001). (2) College students’ perceived social support partially mediated the relationship between health behaviors and learning engagement. Conclusion: One of the main ways to improve college students’ learning engagement is to improve their health behavior and perceived social support. This study contributes to a better understanding of the relationships between health behaviors and learning engagement, as well as to the development of interventions to improve learning engagement among college students.

## 1. Introduction

### 1.1. The Raising of Questions

The Internet and information technology have accelerated the digitalization of schools, courses and telemedicine [1]. Due to the sudden epidemic of COVID-19, which limits people’s communication and interaction and makes face-to-face teaching difficult, online education has become the mainstream teaching method in schools [2]. However, being in the network environment for an extended period of time will cause students to experience psychological distress and negative emotions such as anxiety, boredom and so on [3]. It reduces learning motivation and cognitive investment in the learning process [4]. Therefore, in the context of COVID-19 prevention and control, how to improve students’ learning engagement has become a problem worth considering.

Learning engagement is a learning state characterized by focus, dedication and energy, in which learners can devote themselves to their learning tasks without becoming easily exhausted. Current knowledge about the origin of learning engagement varies, but most scholars use the concept of “time on task” introduced by American educator Ralph W. Tyler as a starting point to explore learning engagement. In his book “Fundamentals of Curriculum and Instruction”, Tyler pointed out that the essence of learning is an active behavior that occurs as a result of the learner’s own response to the environment he or she is in. Pace proposed the “quality of effort” in 1982 [5], and together with Benjamin Bloom, developed a questionnaire to measure the “quality of effort” in college students’ learning experiences. In 2002, Schaufeli extended the study of work engagement by shifting the study population to students, extending work engagement to learning engagement and providing a psychological perspective on learning engagement. They viewed learning engagement as a cognitive state that can be measured in terms of motivation, energy and concentration [6]. High levels of academic engagement can lead to increased academic satisfaction [7]. It also improves physical and mental health while decreasing the likelihood of students dropping out [8]. Low academic engagement in adolescents, on the other hand, leads to academic failure and unhealthy behaviors [9].

Kasl introduced the concept of health behavior in 1966, which defined health behavior as an individual’s action to prevent or detect disease early [10]. In 1986, Duffy pointed out in a survey of health behavior that health behavior consists of both conscious and unconscious behaviors that are beneficial to health [11]. With this definition, we can see that scholars have recognized the role of the individual’s cognition or consciousness in health behavior, and the idea that cognitive and affective aspects have become important components of health behavior has become more widely accepted. There is a link between healthy behaviors and learning engagement, according to the literature: appropriate healthy behaviors can increase students’ learning engagement [12].

The term “social support” was introduced by Cassel and Cobb [13] in 1976 in the psychiatric literature. There are two main types of social support: observed support, also known as action support; and subjective experiential support, also known as perceived support, which is the level of emotion and satisfaction felt by individuals while feeling respect, support and understanding or while apprehending social support—that is, how individuals perceive and assess the level of support they receive from family, friends and significant others [14]. Positive learning motivation can be obtained when individuals perceive high levels of external support and expectations [15]. The intensity of learning motivation has an important impact on students’ academic engagement [16]. Social support is a positive social psychological factor, which is closely related to health behaviors and can improve self-behavior [17].

According to the existing studies, learning engagement is related to health behaviors and perceived social support to varying degrees, but few studies link the three in a unified framework. Valentina Cesari discovered the role of flow, engagement and social interactions during online sessions can cope with distractions and boredom during online learning [18]. Therefore, in order to further study and solve the problem of the impact of learning engagement in the process of online education, this project takes college students in Henan Province of China as the survey object to investigate the learning engagement, health behavior and perceived social support of college students during the epidemic period; clarify the relationship between health behaviors, perceived social support and learning engagement; and explore the internal mechanisms of their interactions. The aim is to further improve the health behaviors of college students and enhance perceived social support; to make college students have positive attitudes towards learning issues; and finally, to increase the level of learning engagement.

### 1.2. Research Hypothesis

Self-determination theory is a theory related to human motivation. Deci and Ryan pointed out that an individual’s intrinsic resources and social environment are both determined by the individual’s motivation and environment [19]. This study explores the relationship between college students’ health behaviors, understanding social support and learning engagement, all of which are inseparable from the role and influence of personal, family, school, society and other environments, which aligns with the self-determination theory viewpoint.

There is a significant correlation between health behaviors and social support. Health behavior is a complex manifestation that is influenced by many factors, including personal, social and environmental factors. According to research, perceived support plays an important role in promoting positive health behaviors [20], and adolescents who receive more support from parents and friends are more likely to engage in physical activity and maintain their health intentions and habits [21]. Regular participation in sports activities can not only broaden one’s social network, but also improve one’s ability to accept and provide social support. Physical activity is a critical component of increasing people’s social support [22].Based on this, this paper proposes hypothesis H1: The health behaviors of college students have a positive effect on the degree of perceived support of college students.

Social support is vital in the process of securing learning engagement. Guichun Jin et al. found that schools can make students more grateful and invest more in learning by strengthening teachers’ emotional support for teenagers and meeting students’ basic psychological needs [23]. Ying Zhao and others confirmed that social support is a key factor in improving academic youth. Some parents and educators should build an effective social support system, improve students’ perception of social support and enhance student learning [24].Based on this, this paper proposes hypothesis H2: the social support of college students’ understanding has a positive influence on the level of students’ learning engagement.

Adolescents’ participation in healthy behaviors such as sports is an important way to boost the organism’s immunity. A healthy body is the guarantee of learning. At the same time, regular physical exercise can stimulate the development of the brain, improve cognitive function, improve attention and so on [25]. Yifei Gao investigated the status of family health behaviors and learning engagement in the new crown pneumonia epidemic [26]. The analysis of factors affecting physical activity among college students revealed a significant positive relationship between physical activity, learning engagement and self-esteem; that adolescents’ athletic ability had a positive predictive effect on their learning engagement; and that physical activity had an effect on the learning engagement of middle school students.In view of this, this paper proposes hypothesis H3: The health behaviors of college students have a positive effect on students’ learning engagement levels.

**Figure 1:**
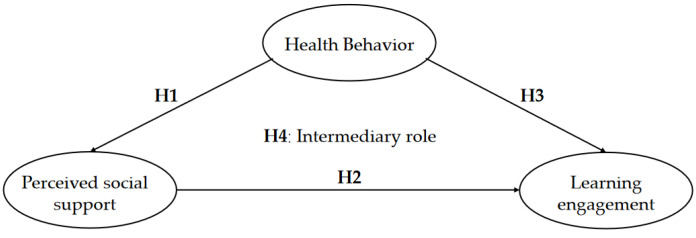
The hothesized model.

Based on the existing literature, health behaviors can influence social support, and social support can also play a role in learning engagement. Therefore, hypothesis H4: College students’ perceived social support has a partially mediating role in the relationship between health behaviors and learning engagement. The hothesized model is shown in Figure 1.

## 2. Research Methodology

### 2.1. Research Subjects

The survey was conducted in Henan Province, China from 15 April 2022 to 20 May 2022. In order to reduce sampling error, stratified cluster sampling was used to ensure the diversity of participants. Two universities were chosen based on the current epidemic situation in Henan Province. First, investigators were recruited from the two universities, and they received standardized training. The investigator then forwarded the digital questionnaire to the participants via a link, and they answered via the Questionnaire Star platform, which is widely used in China. The questionnaire was conducted in Chinese. Participants respond by clicking on the link, and the questionnaire is automatically collected after submission. A total of 589 questionnaires were collected, 538 of which were valid, yielding a 91.3% effective rate. Inclusion criteria: (1) Respondents read the informed consent form and volunteered to participate in the study; (2) completed the online questionnaire on their own or with the help of investigators; (3) have basic cognitive ability to understand the meaning of each item in the questionnaire. Exclusion criteria: (1) Non-native language participants; (2) questionnaires with conflicting information. Detailed statistical data of the subjects are shown in Table 1.

### 2.2. Research Tools

To ensure the questionnaire’s scientificity and validity, it was divided into two sections: basic information (including basic family information, personal information and epidemic situation) and standard scale (including health behavior scale, Perceived Social Support Scale and Learning Engagement Scale).

(1) Health Behavior Scale (HBS)

This scale, developed by Yunlong Lu [27], consists of two dimensions—health intentions (3 items) and health habits (3 items)—and each item is scored on a 5-point Likert-type scale, with higher scale scores indicating a lower level of health behavior among subjects during the epidemic.

(2) Perceived Social Support Scale (PSSS)

In this paper, we refer to the revised Perceived Social Support Scale translated by Qianjin Jiang et al. [28]. The main change in this paper is to replace “leaders, relatives, and colleagues” with “teachers and classmates”. The scale consists of 12 items, which can be divided into three areas: family support, friend support and other support. Each item is rated on a 7-point Likert-type scale, and the higher the total score, the higher the level of social support.

(3) The Utrecht Work Engagement Scale—Student

This scale was developed by Schaufeli and other scholars. There were 17 questions, including three dimensions: vigor, dedication and absorption. Then it was revised by Xiying Li [29], he replaced “work” with “learning” in the original scale and named the three dimensions of the scale. Each item is scored on a 7-point Likert-type scale, and the higher the total score, the higher the level of learning engagement.

### 2.3. Data Analysis Method

In this paper, CFA single-factor test and multiple-factor test were used to test the common method deviation. IBM SPSS Statistics 26 (IBM SPSS Inc., Chicago, IL, USA) was used to analyze the correlation and difference of each variable. The t-test was used to compare groups’ health behavior, perceived support degree and learning engagement. Gpower (Heinrich Heine University, Dusseldorf, Germany) was used to calculate the effect size and power level. IBM SPSS Amos 26.0 (IBM SPSS Inc., Chicago, IL, USA) was used to analyze the reliability and validity of the scale, and a structural equation model was constructed to determine the direct path affecting learning and the mediating effect of perceived social support on learning engagement through health behaviors. All tests were two-tailed, and the significance level was set at 0.05.

## 3. Research Results

### 3.1. Testing of Research Instruments

(1) Common method bias

To test for possible common method bias, this paper uses the CFA single-factor and multifactor tests. For single-factor fitness analysis, all question items were placed within the same factor, and for multifactor fitness analysis, each question item was placed within the factor to which it belonged. The fitness of the single-factor model and the multifactor model [30] was examined, and if the indicators between them were very different, it meant that the two models were different and it could be concluded that the model did not have a high degree of common method bias.

**Figure 2:**
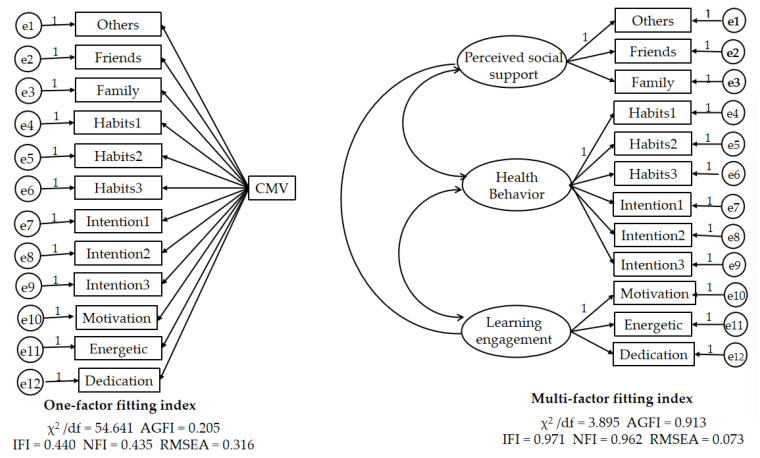
Common method deviation test. Note: e1–e12 are residual terms.

Figure 2 depicts the results of fitting the single-factor and multifactor models, and the results indicate that there is a significant difference between the single-factor fitting indicators and the multifactor fitting indicators, implying that there is no high common method bias in this study model, and the constructed model has some practical significance.

(2) Reliability test

This reliability analysis was completed by IBM SPSS Amos 26.0, and the results of the analysis are shown in Table 2. The reliability analysis was performed using the test proposed by Guieford in 1998; that is, a Cronbach’s alpha coefficient higher than 0.75 indicates a high reliability of the question items. As shown in Table 2, the standardized factor loadings of each measurement item were greater than the standard value of 0.5, and the Cronbach’s alpha coefficient of each scale was greater than 0.9, indicating that each measurement scale had good reliability. The convergent validity of the measurement scales was also examined, using the indicator evaluation criteria of Hairs et al. [31], with combined reliability (CR) and average variance extracted (AVE) as judgment indicators, and the results showed that CR was 0.902–0.967 and AVE was 0.605–0.907, and all indicators met the criteria (CR > 0.7, AVE > 0.5), indicating that the convergent validity of each measurement scale was good.

(3) Correlations between different variables and differences

As shown in Table 3, the correlation coefficients between the variables were 0.286–0.491, and the correlation between the variables was significant (*p* < 0.01). At the same time, the correlation coefficients between each variable were less than the square root of AVE, indicating good discriminant validity between the scales [32].

The presence or absence of new cases at the school location was used as the independent variable, and the variables were analyzed for variance using the statistical software IBM SPSS Statistics 26. Considering that the categorical variables were dichotomous, the independent samples *t*-test was used, and the test results are shown in Table 4 below.

The mean values of academic engagement, perceived social support and health behavior of college students during the epidemic were 4.6045, 5.2752 and 3.8386, respectively, with standard deviations of 1.1825, 1.0742 and 0.6029, indicating that college students had average levels of academic engagement, good levels of perceived social support and average levels of health behavior during the epidemic. As shown in Table 4, there were significant differences in the dimensions of learning engagement among college students based on the presence or absence of new cases at the school location, with students who did not have new cases at the school location having significantly higher levels of motivation, energy and concentration than students with new cases (*p* < 0.001). The effect size and power level were tested by Gpower, and Cohen’s D of each dimension was close to the medium level (0.5) and the statistical power was good (>0.8). There were significant differences in the dimensions of perceived social support among college students on the factor of presence or absence of new cases at the school location (*p* < 0.05). The most significant difference was friends’ support (*p* < 0.01), and the power level was 0.854 (>0.8). There were significant differences in health intentions and no significant differences in health habits among students with or without new cases at school location. Among them, the level of health intention was higher in schools with no new cases in the school location (*p* < 0.05), and the power level is 0.657 (>0.5).

(4) Testing of structural equation model

IBM SPSS Amos 26.0 was used to construct SEM structural equation models of the direct effect of health behaviors on learning engagement and the mediating effect of comprehending social support in the influence of health behaviors on learning engagement. In order to make the model fit the data better, this model used the topic packing strategy of Yan Wu and Zhonglin Wen (2011) [33] to measure the two variables of apprehending social support and learning engagement, which were packaged to form new observations. This study evaluates the overall fit of the SEM structural model in terms of its absolute fit, value-added fit and parsimonious fit, and Table 5 shows the results of the structural equation model fit test.

**Figure 3:**
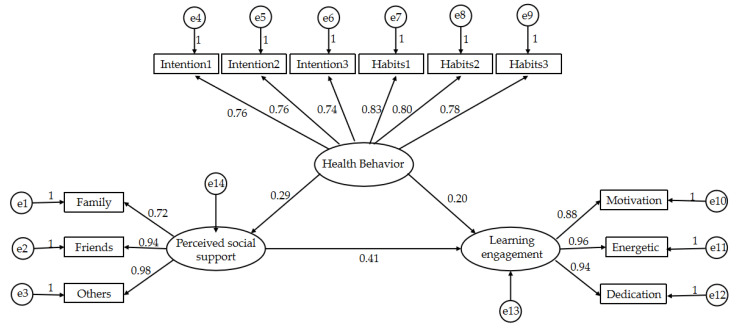
Model of the relationship between health behavior, comprehending social support and learning engagement. Note: e1–e14 are residual terms.

As shown in Table 5, the absolute fitness evaluation indexes are: χ^2^ = 1980.629, GFI = 0.943, AGFI = 0.913, RMSEA = 0.073; value-added fitness evaluation indexes are: NFI = 0.962, IFI = 0.971, CFI = 0.971; and parsimonious fitness evaluation indexes are: χ^2^/df = 3.895, PNFI = 0.743, PGFI = 0.617. The actual values of the model fitness indicators were compared with the ideal values, and the actual values of the reported fitness indicators were within the recommended range. It can be seen that the present model assumptions fit well with the actual data and the model settings can be accepted. The verified model is shown in Figure 3.

### 3.2. Structural Equation Model Analysis

(1) Path coefficient estimation and hypothesis testing

Table 6 depicts the relationships between the three latent variables in the measurement model and their observed variables, and the analysis leads to the following conclusions:

① In this study, the three observed variables of perceived social support—family support, friend support and other support—can all reflect perceived social support, and the standardized path coefficients between these observed variables and perceived social support are 0.725, 0.940 and 0.984, all of which are positive, indicating that in terms of college students’ perceptions of social support, the higher the degree of support that students receive from family, the stronger the support from friends and the more extensive other external support, the higher the perceived social support.

② In this study, the three observed variables of learning engagement—motivation, energy, and concentration—can all reflect learning engagement, and the standardized path coefficients between these observed variables and learning engagement are 0.879, 0.962 and 0.945, and all of them are positive, indicating that the higher motivation, energy and concentration are, the higher the level of college students’ learning engagement is.

③ The following six variables of health behavior were observed in this study: the need for physical exercise to satisfy their own life and study; the degree of their interest and hobby in physical exercise; how the atmosphere of physical exercise is in their school; whether they will overcome some inertia for physical exercise; how they feel the effect of exercise and emotional experience after physical exercise; and whether they think physical exercise and the value of physical exercise are important to the health behavior of college students. The standardized coefficients were 0.833, 0.798, 0.740, 0.756, 0.755 and 0.780, respectively.

All the path coefficients passed the significance test, the research hypotheses proposed in the previous paper could be verified, and the hypothesis test results are as follows.

The standardized path coefficients among the variables are shown in Table 7, and the results indicate that: first, college students’ health behaviors have a significant positive effect on college students’ perceived social support (β = 0.289, *p* < 0.001); second, college students’ perceived social support has a significant positive effect on students’ learning engagement levels (β = 0.200, *p* < 0.01); and third, college students’ health behavior has a significant positive effect on students’ learning engagement levels (β = 0.406, *p* < 0.001). Therefore, the hypotheses H1, H2 and H3 of this study are valid.

(2) Test of mediating effect of comprehending social support

**Figure 4:**
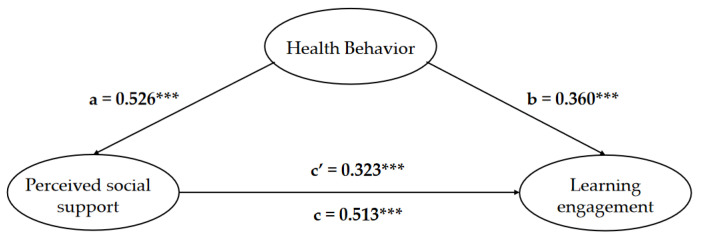
Analysis of the relationship between health behavior, comprehending social support and learning engagement (mediating effect). Note: *** *p* < 0.001, c = a ∗ b + c′.

In order to more visually describe the relationship between apprehending social support, health behavior and learning engagement, the results of the path relationship between the three variables are represented in Figure 4. The direct effect of health behavior on perceived social support was 0.526; the direct effect of perceived social support on learning engagement was 0.360; the direct effect of health behavior on learning engagement was 0.323; and the total effect of health behavior on learning engagement was 0.513.

The bootstrap method was used to conduct the mediating utility test in order to investigate the significance of the mediating role of potential variables in the structural model of the substudy. Table 8 shows the result of 5000 replicate sampling tests at 95% confidence intervals to find the standardized coefficients of the paths between the variables in this study and their standard errors using the great likelihood method.

The results of the mediating effect test between the variables were also obtained (Table 9). If the 95% confidence interval of both the bias-corrected method and percentile method in the bootstrap test does not contain 0, the effect is significant.

The results are shown in Table 9: the total effect value of this mediated model is 0.513 and the total effect of this model is significant in the bootstrap test with 5000 repetitions of sampling in both the bias-corrected method and percentile method with the 95% confidence interval not containing 0. The direct effects a and b of the model were tested, and both passed the significance level test, indicating that the perceived social support of college students plays a mediating role between health behavior and learning engagement. In addition, the indirect effect value of perceived social support in the relationship between health behavior and learning engagement was 0.190, and the 95% confidence intervals of both BC and PC did not include 0, indicating that perceived social support of college students plays a partially mediating role between health behavior and learning engagement. Therefore, the perceived social support of college students plays a partially mediating role between health behaviors and learning engagement, and hypothesis H4 holds.

## 4. Analysis and Discussion

### 4.1. Current Situation and Characteristics of College Students’ Learning Engagement, Perceived Social Support and Health Behaviors during the Epidemic

In this study, we found that college students’ study engagement level was not high, and that students in schools with new cases had lower study engagement than students in schools without new cases. Because online teaching replaces the original traditional teaching, effective digital infrastructure and digital skills of students and teachers are required, and limited classroom interaction significantly affects the satisfaction of students and decreases the level of students’ learning engagement. Kunal Chaturvedi conducted a survey on the impact of COVID-19 on students’ education, social life and mental health. The results showed that the amount of time students spent on online courses did not meet the requirements set by the Ministry of Education, which confirmed that COVID-19 had a certain impact on students’ learning engagement [34].

In general, college students reported a high level of perceived social support. The presence or absence of new cases at school locations had effects on the dimensions of perceived social support, with the dimensions of friend support being significantly reduced. COVID-19 has plunged the world into an unprecedented public crisis. In order to limit the spread of the virus, reducing social gatherings, maintaining social distancing and isolation have become the main prevention and control measures. During the outbreak period, face-to-face communication and interaction could only be limited to core grid personnel with close relationships, such as family members and cohabiting roommates, which weakened social relationships and provided diversified resources and support of support [35], lowering the perceived social support level of students at school.

Among the health behaviors of college students, physical exercise of college students mainly focuses on small and medium exercise levels, and in terms of physical exercise intensity, the majority of them choose to exercise at less than medium intensity. However, there was no significant difference in health habits. The level of students’ health intention in schools with new cases recently is lower, which indicates that the outbreak of the epidemic has a certain impact on students’ health intention and that some college students are not willing to choose to go outdoors for some healthy exercise activities in order to avoid risks. Leandy Bertrand also confirmed inactivity and increased sedentary behavior among students during the pandemic [36].

### 4.2. The Relationship between Social Support, Health Behaviors and Learning Engagement among College Students during an Epidemic

(1) As shown in the previous section, there were significant positive relationships between health behavior and perceived social support, health behavior and learning engagement, and perceived social support and learning engagement. The results showed the health behaviors of college students (β = 0.289, *p* < 0.001). Previous studies have confirmed the positive effect of social support on health behaviors such as sports [37,38], and our study found that these health behaviors also promote students’ perceived social support. The COVID-19 epidemic has brought a huge loss of resources and risks to people’s lives, as well as heavy mental pressure and distress to people [39]. Healthy behaviors may have promoted prosocial motivation and better emotional adjustment [40], enhance students’ self-construction of physical and mental health [41] and improve their subjective perception ability so that individuals can more easily perceive support, respect and other emotional experiences from the external environment. This strengthens and promotes the understanding of social resources and the perception of family, friends and other social support. (2) Perceived social support had a positive effect on learning engagement (β = 0.200, *p* < 0.01), indicating that the higher the perceived degree of social support, the higher the level of learning engagement of college students. The sources of social support that students receive can be generally divided into three aspects: family and parents, school and teachers and other peers. From the point of view of the family, the participation of and support from their parents as well as strong family cohesion [42] can let them feel warm and valued, and students’ academic attitude, faith and recognition of the importance of education will strengthened to a certain extent. Thus, a higher level of learning engagement can be predicted. From the perspective of schools, schools and teachers provide students with basic psychological support and support for autonomous needs, so that students feel care and expectation, their intrinsic motivation to participate in learning is activated [23] and their sense of learning self-efficacy is also enhanced [24], thus promoting students’ high levels of investment in learning. From the perspective of peers, through the perception of positive guidance and support from others, students internalize it and integrate it into their own learning participation process [43], thus enhancing their learning involvement intensity. (3) During the epidemic period, the health behaviors of college students had a positive impact on the learning engagement of college students (β = 0.406, *p* < 0.001). Regular physical exercise can not only strengthen the body, but also disperse the pressure caused by negative emotions and promote mental health [44]. Under the control of positive emotions, it can improve the working ability of the brain, enhance the activity of thinking and then increase the level of learning engagement of college students. Zhang Xiao et al. also reached a similar conclusion that physical exercise was closely related to learning engagement of college students, and physical exercise health behaviors could positively predict learning engagement. (4) Perceived social support of college students during the epidemic played a partial mediating role between health behaviors and learning engagement. The mediating analysis results showed that health behavior could significantly predict students’ learning engagement level before perceived social support was included in the analysis; when perceived social support was included in the analysis, the effect of health behavior on students’ learning engagement level was still significant, indicating that perceived social support played a partial mediating role between the two. During the COVID-19 pandemic, students’ psychological stress gradually increased, which has led to a significant decrease in learning engagement. Students’ recognition of completing certain tasks has decreased, which can also directly or indirectly affect health behaviors. In addition, regular participation in physical activity is beneficial to college students’ psychological and cognitive development; thus, there is more recognition and acceptance of social support and the various social aspects to make a higher evaluation [45]. Students gain positive social support, thereby enhancing responsibility, achievement and satisfaction, as well as contributing to the formation of positive emotions and temperament.

## 5. Limitations of the Study

The results of this study have some implications for educational practice and help to understand the factors and mechanisms affecting students’ learning engagement, but there are also some limitations. First of all, the study selected college students as subjects, without making detailed distinctions according to grades and majors. Students at different stages and majors may feel different sources of social support. In addition, we found in this study that college students’ health habits were not affected in the context of epidemic prevention and control. In order to further confirm, it is necessary to increase the sample size and further explore by learning stages and majors. Secondly, this study only considered the influence of social support and health behavior on learning engagement, and the influencing factors of learning engagement include learning self-efficacy, learning satisfaction, learning value, etc. [46]. The influence and mechanism of these factors on learning engagement need to be further studied and analyzed.

## 6. Conclusions and Suggestions

The preceding analysis and discussion lead to the following conclusions: First, there were significant positive correlations among health behaviors, perceived social support and learning engagement of college students. The higher the level of health behavior of college students, the stronger their ability to perceive social support; the perceived social support ability of college students is helpful to improve the level of college students’ learning engagement. At the same time, the level of health behavior of college students has a positive impact on their level of learning engagement. Second, perceived social support of college students partially mediates the relationship between health behaviors and learning engagement; that is, the direct effect of health behavior on students’ learning engagement level is significant, and the indirect effect of health behavior on students’ learning engagement level is still significant after the analysis of perceived social support participation. Third, improving college students’ health behaviors and their ability to perceive social support are important ways to increase their learning engagement.

Combining the above findings, the following suggestions were made to improve learning engagement.

(1) School level: Schools can organize regular recreational and sports activities in which students can participate to alleviate the negative emotions caused by the long-term closure of school and improve students’ learning engagement. In addition, the school can also build an online learning communication platform to facilitate learning exchanges between teachers and students, and timely online feedback from teachers can increase students’ learning experience.
(2) Teacher level: Including sign-in and questioning during online classes can improve students’ classroom participation by guiding their active participation; at the same time, teachers should provide students with full support and affirmation so that students can learn in a pleasant environment and boost their learning confidence, thereby improving students’ motivation in class.
(3) Student level: When performing physical exercise, they should pay attention to choosing the appropriate exercise intensity and exercise time to avoid blindly pursuing the exercise effect and neglecting their physical condition, which can damage their health.

## Figures and Tables

**Table 1:** Results of statistical analysis of subjects (*n* = 538).

Variables	Categories	Number	Effective Percentage (%)
Gender	Male	279	51.9
	Female	259	48.1
Home location	Urban	152	28.3
	Rural	386	71.7
Grade	Freshman	93	17.3
	Sophomore	134	24.9
	Junior	50	9.3
	Senior	261	48.5
Type of specialization	Science and Engineering	118	21.9
	Others	340	63.2
	Literature and History	80	14.9
Classes	Online	132	24.5
	Online and offline	299	55.6
	Offline	107	19.9
Any new cases at the school site	Yes	240	44.6
	No	298	55.4

**Table 2:** Scale reliability and convergent validity analysis.

Scale	Variables	Standardized Factor Loadings						CR	AVE	α
		Q1	Q2	Q3	Q4	Q5	Q6			
Learning Engagement	Motivation	0.888	0.807	0.846	0.917	0.903	0.879	0.967	0.907	0.981
	Energy	0.898	0.885	0.886	0.941	0.922	0.936			
	Dedication	0.914	0.939	0.917	0.898	0.909	-			
Perceived Social Support	Family	0.778	0.895	0.874	0.811	-	-	0.940	0.840	0.962
	Friends	0.937	0.921	0.934	0.908	-	-			
	Others	0.875	0.908	0.879	0.921	-	-			
Health Behavior	Intention	0.832	0.799	0.737	-	-	-	0.902	0.605	0.901
	Habits	0.756	0.758	0.781	-	-	-			

**Table 3:** Results of correlation analysis.

Variables	M	SD	Perceived Social Support	Learning Engagement	Health Behavior
Perceived Social Support	5.2752	1.07415	0.916		
Learning Engagement	4.6054	1.18251	0.491 **	0.952	
Health Behavior	3.8386	0.60291	0.286 **	0.319 **	0.778

Note: ** *p* < 0.01.

**Table 4:** Variance analysis report table.

Variables		Any New Cases at the School Site		*t*-test			
		Yes	No	*t*	*p*	Cohen’s d	1-β
Learning Engagement	Motivation	4.689 ± 1.049	5.054 ± 1.197	−3.769	<0.001	0.324	0.962
	Energy	4.106 ± 1.240	4.665 ± 1.313	−5.029	<0.001	0.438	0.999
	Dedication	4.184 ± 1.215	4.736 ± 1.265	−5.121	<0.001	0.445	0.999
Perceived Social Support	Family	4.991 ± 1.207	5.244 ± 1.225	−2.401	0.017	0.208	0.668
	Friends	5.205 ± 1.091	5.498 ± 1.147	−3.011	0.003	0.262	0.854
	Others	5.192 ± 1.095	5.436 ± 1.189	−2.456	0.014	0.213	0.69
Health Behavior	Intention	3.753 ± 0.583	3.880 ± 0.652	−2.394	0.017	0.205	0.657
	Habits	3.825 ± 0.600	3.870 ± 0.662	−0.826	0.409	0.071	0.13

**Table 5:** Evaluation table of the fitness index of the structural equation model.

Evaluation Indicators	Indicators	Ideal Value	Actual Values	Standard Sources
Absolute fitness evaluation	χ^2^	As small as possible	198.629	Ming-Lung Wu: Structural Equation Modeling (2009)
	GFI	>0.90	0.943	
	AGFI	>0.90	0.913	
	RMSEA	<0.08	0.073	
Value-added suitability evaluation	NFI	>0.90	0.962	
	IFI	>0.90	0.971	
	CFI	>0.90	0.971	
Minimalist suitability evaluation	χ^2^/df	<5	3.895	
	PNFI	>0.50	0.743	
	PGFI	>0.50	0.617	

**Table 6:** Table of path estimation for each variable.

Path Analysis			Estimate	S.E.	C.R.	St. Estimate
Perceived Social Support	←	Health Behavior	0.526	0.082	6.419 ***	0.289
Learning Engagement	←	Health Behavior	0.323	0.07	4.621 ***	0.200
Learning Engagement	←	Perceived Social Support	0.36	0.038	9.572 ***	0.406
Others’ Support	←	Perceived Social Support	1			0.984
Friends’ Support	←	Perceived Social Support	0.937	0.021	43.743 ***	0.940
Family Support	←	Perceived Social Support	0.781	0.034	22.754 ***	0.725
Motivation	←	Learning Engagement	1			0.879
Energy	←	Learning Engagement	1.249	0.035	35.915 ***	0.962
Dedication	←	Learning Engagement	1.191	0.034	34.719 ***	0.945
To meet their own life study needs physical exercise?	←	Health Behavior	1			0.833
The degree of their interest and hobby in physical exercise	←	Health Behavior	0.931	0.044	21.244 ***	0.798
How is the physical activity atmosphere in their school?	←	Health Behavior	0.855	0.045	19.137 ***	0.740
Will they overcome some inertia for physical exercise?	←	Health Behavior	0.902	0.046	19.705 ***	0.756
How do they feel about the exercise effect and emotional experience after physical exercise?	←	Health Behavior	0.939	0.048	19.671 ***	0.755
Do they think physical activity and the value of physical activity is important?	←	Health Behavior	0.922	0.045	20.555 ***	0.780

Note: *** *p* < 0.001.

**Table 7:** Path hypothesis tests among the variables.

Research Hypothesis	Path Relationships	St. Estimate (β)	*p*	Test Results
H1	Health Behavior → Perceived Social Support	0.289	***	Yes
H2	Perceived Social Support → Learning Engagement	0.200	**	Yes
H3	Health Behavior → Learning Engagement	0.406	***	Yes

Note: ** *p* < 0.01, *** *p* < 0.001.

**Table 8:** Table of standardized estimates and their standard errors reported.

Paths			SE	SE–SE	β	Bias	SE-Bias
Perceived Social Support	←	Health Behavior	0.060	0.001	0.289	0.001	0.001
Learning Engagement	←	Health Behavior	0.055	0.001	0.200	0.002	0.001
Learning Engagement	←	Perceived Social Support	0.053	0.001	0.406	−0.003	0.001

**Table 9:** Reported results of mediating effect test.

Effect Categories	Coefficient Multiplication Product		95% Confidence Interval			
	S.E.	β	Bias-Corrected Method		Percentile Method	
			Lower	Upper	Lower	Upper
Direct effect a	0.060	0.289	0.162	0.402	0.167	0.407
Direct effect b	0.055	0.406	0.295	0.505	0.292	0.502
Direct effect c′	0.053	0.200	0.089	0.303	0.092	0.308
Intermediary effect (ab)	0.036	0.190	0.126	0.266	0.117	0.257
Total effect	0.099	0.513	0.309	0.701	0.314	0.705

## Data Availability

Data are available, upon reasonable request, by emailing: yingjikexue@hpu.edu.cn.
